# Synthesis and Properties of Water-Based Acrylic Adhesives with a Variable Ratio of 2-Ethylhexyl Acrylate and n-Butyl Acrylate for Application in Glass Bottle Labels

**DOI:** 10.3390/polym12020428

**Published:** 2020-02-12

**Authors:** Irene Márquez, Felipe Alarcia, José Ignacio Velasco

**Affiliations:** 1 Lubrizol Advanced Materials, Applications Department, Camí de Can Calders, 13, 08173 Sant Cugat del Vallès, Spain; Irene.Marquez@lubrizol.com (I.M.); Felipe.Alarcia@lubrizol.com (F.A.); 2 Department of Materials Science and Engineering, Universitat Politècnica de Catalunya (UPC BarcelonaTech), ESEIAAT, Carrer de Colom, 11, 08222 Terrassa, Spain

**Keywords:** acrylic PSA, water-borne adhesives, glass bottle labels

## Abstract

A series of Pressure-Sensitive Adhesives (PSAs) with different soft monomer compositions were prepared by using emulsion polymerization. The monomers used were acrylic acid (AA), n-butyl acrylate (n-BA) and 2-ethylhexyl acrylate (2-EHA). Maintaining the same acrylic acid fraction in all polymerizations, the n-BA/2-EHA weight ratio varied from 0 to 1. These polymers were characterized by using Fourier Transformed Infrared Spectroscopy (FTIR), and the Glass Transition Temperature (*T*_g_) was determined both theoretically from the Fox equation and experimentally by means of differential scanning calorimetry (DSC). The tetrahydrofuran (THF) insoluble polymer fraction was used to calculate the gel content, and the soluble part was used to determine the average molecular weight by means of Gas Permeation Chromatography (GPC). The adhesive performance was assessed by measuring tack, peel and shear resistance. The results showed that with the 2-EHA rate, the elastic modulus slightly decreased and the shear yield strength slightly increased. Consequently, the loop tack and peel resistances decreased. This behavior was attributed to the increase of the gel content with the ratio of comonomers studied. The adhesives were tested in paper labels on glass bottles immersed in a cold-water bath, the so-called ice bucket test, and all of them showed that they could withstand wet and cold environment conditions.

## 1. Introduction

One of the polymers most used in adhesives for glass bottle labels is casein. Casein, of animal origin, is very susceptible to strong fluctuations in price and quality and can easily suffer bacterial contamination. In addition, the casein-based adhesives are applied in wet conditions, that is, the adhesive is applied just before the label is going to be stuck to the bottle [1]. This is a disadvantage when it comes to facilitating the process and therefore, there is a great interest in replacing them by Pressure-Sensitive Adhesives (PSAs) [2]. PSAs are an interesting class of viscoelastic materials that are characterized by their ability to adhere strongly to a wide variety of substrates at room temperature with the application of slight pressure for a short period of time [3]. Furthermore, unlike casein, PSAs allows labels to be made and stored in reels, which clearly improves the labeling process and its efficiency.

Acrylic adhesives are an interesting class of PSAs with a high growth in the labels market, due to their high molecular weight and low *T*_g_, among other features. Between the different methods, the emulsion polymerization offers great advantages such as a low cost, low viscosity products independently of the polymer molecular weight, high solid content as well as environmental safety due to the use of water as a solvent [3,4,5]. One of the disadvantages of emulsion-based PSAs is that their water resistance tends to be weak. Obviously, this can be an important disadvantage, as a label must have a certain water resistance since they must be able to withstand certain humidity conditions [6]. There are different ways to increase the water resistance of a water-based polymer. One of them is to use monomers with a low water solubility.

The n-butyl acrylate (n-BA) and the 2-ethylhexyl acrylate (2-EHA) are two of the most used soft monomers for water based acrylic PSAs. Because of their low *T*_g_ (−54 and −70 °C, respectively) [7] and low water solubility (0.15 g/100 cm^3^ and 0.04 g/100 cm^3^ at 25 °C) [5], they need to be copolymerized with some hard monomers, such as acrylic acid (AA) (*T*_g_ = 105 °C) [8,9]. There are several studies that show that after increasing the amount of 2-EHA monomer, the *T*_g_ of the resulting polymer decreased. Moreover, it has higher tendency to generate gel. The gel content was defined as the fraction of polymer that is not soluble in tetrahydrofuran (THF) at reflux (70 °C). When we compared poly(butyl acrylate-co-acrylic-acid) to poly(2-ethylhexyl acrylate-co-acrylic acid), the adhesive performance was found to be related to the gel content. Due to its molecular structure, it is known that 2-EHA is less water soluble [5] and more favorable to form gel than n-BA [10]. In another study, Moghbeli, Zamir and Molaee [7] observed a reduction in gel content with the increase of 2-EHA monomer of up to 50%. However, with higher levels of 2-EHA, they obtained higher gel contents. Tobing and Klein [11] found a correlation between the gel content and the shear resistance. They observed that as gel content decreased, the tack and peel resistance rose. However, Gower and Sanks [12] observed an increase in the shear resistance and a decrease in the peel resistance while maintaining constant tack values. In other cases, although shear performance improved, no significant changes in the peeling and tack properties were observed [7]. In all cases, the behavior was attributed to the greater facility of 2-EHA comonomer to generate entanglement than BA.

The objective of the present paper was to develop PSAs for glass bottle labels. A standard PSA formula based on n-BA and AA monomers was taken as the starting point for the adhesive, and the effect of partially replacing the n-BA by using 2-EHA was studied. The proportion of AA monomer was constant in all polymerizations. Different proportions in the soft monomer composition were prepared to find an optimal balance between the adhesive properties of the synthesized polymers and the wine cellar technical requirements of the labels. The labeled bottle must pass the ice bucket test, which is a typical test for determining the water resistance of the adhesives in order to check that the adhesive is able to withstand wet and cold environments. In addition, the adhesive must show an adhesion capable of breaking the paper when attempting to remove the label. 

The polymers were characterized by using FTIR and values of theoretical and experimental *T*_g_, obtained by DSC, were compared. The average sol molecular weight and the gel content were determined by GPC and soxhlet extraction, respectively. The adhesive properties, such as tack, peel and shear resistance values were also investigated. Finally, the water resistance was performed by the ice bucket test.

## 2. Materials and Methods 

### 2.1. Materials

The AA and n-BA provided by BASF (Ludwigshafen, Germany) and the 2-EHA provided by Dow Chemical (Pampa, Texas) were used as comonomers in the polymerization. Tert-dodecyl-mercaptan (TDDSH) provided by Chevron Phillips Company LP (Tessenderlo, Belgium) was used as a chain transfer agent. The anionic emulsifier (Dowfax^TM^ 2A1) provided by Dow Chemical (Midland, USA) was also used in the polymerization. Ammonium carbonate ((NH_4_)_2_CO_3_) provided by BASF (Ludwigshafen, Germany) was used as a buffer and ammonium peroxide sulfate ((NH_4_)_2_S_2_O_8_) supplied by United Initiators (Pullach, Germany) was used as a thermal initiator. A combination of tert-butyl hydroperoxide (TBHP), Peroxan, and sodium formaldehyde sulfoxylate (Bruggolite^®^ E01), from Brüggemann KG (Heilbronn, Germany), were used as a redox system to reduce free monomer at the end of the polymerization. A 12.5% ammonia solution, provided by Barcelonesa drugs and chemicals (Conella del Llobregat, Spain), was used to neutralize the adhesives. THF, provided by VWR Chemicals (Radnor, USA), was used as a solvent. 

For adhesion tests, a Polyethylene Terephthalate (PET) provided by Polinas (Manisa, Turkey) with corona treatment and Tintoretto qesso ultraWS^TM^ paper provided by Arconvert (Sant Gregori, Spain) were used as substrates to perform the tests.

For the water resistance tests, instead of using neat paper, a typical label consisting of a silicone-coated paper provided by Gascogne Flexible (Dax, France) as release, Tintoretto gesso ultraWS^TM^ paper as substrate, the Doresco^®^ 7331 from Lubrizol (Sant Cugat, Spain), as primer and post coat for metallization, Aluminum at 99.98% purity for the metallization (provided by Umicore (Balzers, Liechtenstein) and the NC 386 CYAN BASE FE gravure ink from Siegwerk (Barcelona, Spain). 

### 2.2. Emulsion Polymerization

All polymers were prepared at 55% of solid content, adjusting the quantity of water to keep this % constant. The polymerizations were carried out by a semi-continuous process in a 2.5 L glass reactor at 87.5 °C with mechanical stirring. The initial charge in the reactor consisted of 0.3 parts of (NH_4_)_2_CO_3_ per 100 parts by weight of monomer (i.e., 0.3 phm), 0.1 phm of emulsifier and a half of the total water. After heating and purging the reactor with N_2_, 0.6 phm of the initiator agent was introduced, followed by a pre-emulsion composed by the monomeric system (Table 1), 0.1 phm of chain transfer agent, 1.2 phm of emulsifier and the remaining water. The pre-emulsion was added at a constant rate of 9.4 mL/min for 3 h.

Finally, the reactor was cooled down to 55 °C and a redox system was added TBHP/*Bruggolite^®^ E01* (0.2 phm/0.2 phm). Post-polymerization was allowed to take place during 4 h. Gas chromatography analysis indicated that the monomer concentration was lower than 700 ppm in all cases.

### 2.3. Latex Characterization

The polymers were filtered through a 150 µm filter and then analyzed to determine their physical-chemical characteristics.

The pH was measured with the pH Meter Basic 20 instrument, calibrated with buffer solutions of pH = 4.01 ± 0.01 and pH = 7.41 ± 0.01 at 25 °C. The solid content was determined with a CEM Smart System 5 microwave oven, which was calibrated with a standard solution of 9% solid content. The average particle size was measured by dynamic light scattering (DLS) with a Zetasizer Nano Series instrument. Samples were prepared by diluting the polymer in deionized water and analyzed at 25 °C, using a detector with a 90° angle. Averages of three measurements were taken. The viscosity was determined at 25 °C by using a programmable Brookfield DV-II+ rotational viscometer for high viscosities and a Brookfield DV-II+ Pro rotational viscometer for low viscosities.

The gel content was defined as the fraction of the polymer that was not soluble in THF at 70 °C after Soxhlet extraction for 24 h. After that, the residue was dried at 80 °C for 5 h in an oven and the gel fraction was calculated using Equation (1), where *W*_1_ was the initial weight of the filter, *W*_2_ was the weight of the filter with the dry polymer and *W*_3_ was the final weight of the filter after extraction [13].

(1) $$\mathrm{Gel}\;\mathrm{content}\;(\%)\;=\;\frac{W_{3}{-W}_{1}}{W_{2}{-W}_{1}}\;100$$

The soluble fraction was used to determine the average molecular weight (Mw) of the polymer by GPC. The device consists of a Waters 2695 separation module and a Waters 2414 refractive index detector equipped with a 5 µm PL Gel Mixed-D column calibrated with polystyrene standards. THF was used as a continuous phase.

The synthetized polymers were analyzed by FTIR (Perkin Elmer Inc., Waltham, USA) with a Perkin Elmer spectrometer under attenuated total reflectance (ATR) configuration over a range of wavenumber of 4000–400 cm^−1^. The spectra were recorded at room temperature on polymer samples previously dried in oven at 80 °C for 5 h.

The *T*_g_ was determined both theoretically and experimentally. The theoretical values were obtained by using Fox’s equation (Equation (2)) with the *T*_g_ value of the respective homopolymer (*T*_gi_) and the weight fraction (*w*_i_) of each comonomer [14].

(2) $$\frac{1}{T_{g}}=\frac{w_{1}}{T_{g1}}+\frac{w_{2}}{T_{g2}}+\frac{w_{3}}{T_{g3}}$$

The experimental values of *T*_g_ were determined by Differential Scanning Calorimetry (DSC) using the equipment DSC 1, STARe, calibrated with an Indium standard. Samples of about 20 mg were initially placed in the crucibles and dried in an oven at 80 °C for 5 h to obtain dry test samples of about 10 mg. These samples were firstly heated at a rate of 20 °C/min from 25 °C to 200 °C, then held for 15 min to 200 °C and cooled to −65 °C at 20 °C/min. After stabilization for 15 min at −65 °C, the second heating was carried out at 20 °C/min up to 200 °C. The *T*_g_ value was obtained from the second heating curve.

### 2.4. Adhesion Tests

The adhesive properties were evaluated through a shear, peel and tack test. Using a motorized laboratory coater (RK K Control Coater provided by Lumaquin S.A., (Montornès del Vallès, Spain) equipped with a bar of 50 µm, 50 g/m^2^ of polymer was applied onto the substrates, which were subsequently dried in the oven for 1 min at 100 °C, leaving a layer of polymer of approximately 25 g/m^2^. Standard sized tapes were cut for each type of test.

**Figure 1:**
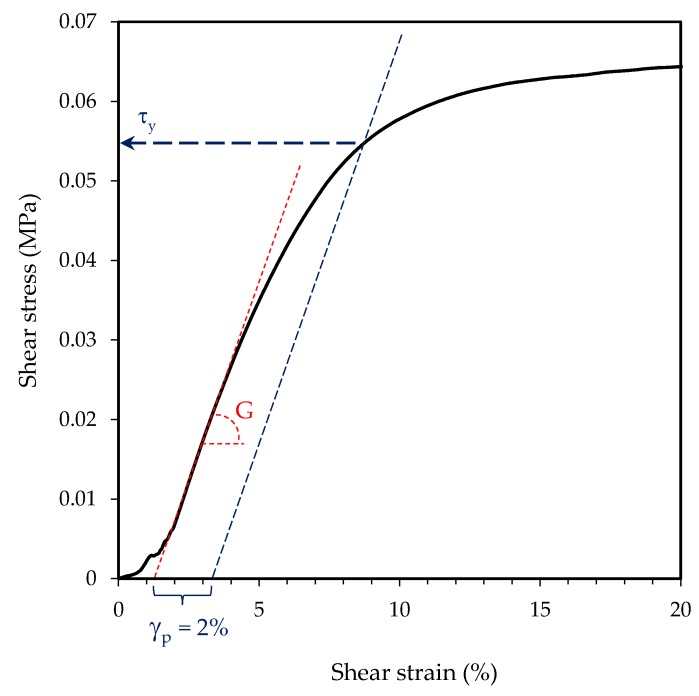
Experimental curve stress vs. strain, registered from dynamic shear testing, with indications of shear modulus (G) and yield stress (τ_y_) determination.

Dynamic shear tests were performed at 5 mm/min with a Zwick/Roell Z 2.5 machine on PET tapes adhered on untreated steel panels at 25 °C with a contact area of 25 × 25 mm^2^. The tape was applied 20 min before the test by means of a rubber roller with a mass of 2 Kg [15]. The shear stress vs. strain curves were recorded and the elastic modulus (G), the yield stress (τ_y_) and the maximum stress (τ_m_) values were determined as shown in Figure 1. The shear modulus was determined as the initial slope of the curve with the linear correlation coefficient (r^2^), which in all cases was higher than 0.99, and the yield stress as the value of shear stress for a plastic shear deformation of 2%.

The peel resistance, defined as the force required to remove a tape from a test panel, was evaluated by means of the 180° peel test after 20 min and 24 h from the tape application. Tapes of PET and paper of 275 × 25 mm^2^ were applied onto glass panels. A Zwick/Roell Z 2.5 tensioner was used at a constant speed of 300 mm/min. The average force to remove the tape and the failure mode were recorded [16]. 

The tack is the capacity of the adhesive to form bonds with a substrate with a brief contact under slight pressure. The tack was determined by the loop tack test with an AT1000 tensile tester equipment. A loop was formed with a PET tape of 175 × 25 mm^2^ and held with the upper clamp. A controlled contact was made at a constant speed of 300 mm/min onto glass panels. The maximum force required to peel off the tape from the panel and the failure mode were recorded [17,18]. 

### 2.5. Water Resistance Tests

Labels of 25 g/m^2^ of each adhesive were built on silicone-coated paper and transferred to the paper. A multilayer barrier coating was designed for the labels. Firstly, the paper was coated with 2.4 g/m^2^ of Doresco^®^ 7331 lacquer to improve adhesion of the aluminum in the metallization process and to keep it in the surface, as otherwise the aluminum would penetrate into the paper. Subsequently, aluminum was metallized by vacuum deposition and another layer of 1.2 g/m^2^ of Doresco^®^ 7331 was applied to protect the metallized layer. The aluminum must be protected in order to avoid its oxidation. Finally, 3.8 g/m^2^ of a gravure ink was applied, covering the whole surface of the label.

The performance of the labels under immersion, condensation and low temperature conditions was determined by the ice bucket test. The labels were stuck on the bottles 24 h before the test to build a stable adhesion on the glass surface [19]. Then, they were placed into an ice and water bath (1:1 by weight) for 24 h. After that, the bottles were removed from the bath and the integrity of the labels was checked. It was determined whether the labels were completely adhered or had some type of imperfection.

## 3. Results and Discussion

### 3.1. Physical-Chemical Properties

Previously to the characterization, all the synthetized adhesives were normalized by adjusting the pH to 7.5 and the solid content to 50 wt.% by neutralizing with ammonia solution (12.5%) and adding deionized water, as necessary.

The particle size and viscosity data obtained are shown in Table 2. In general, the monomer fraction change did not show significant variations in particle size. Although the mean particle size values are not significantly different, a narrow distribution would lead to a higher viscosity. 

**Figure 2:**
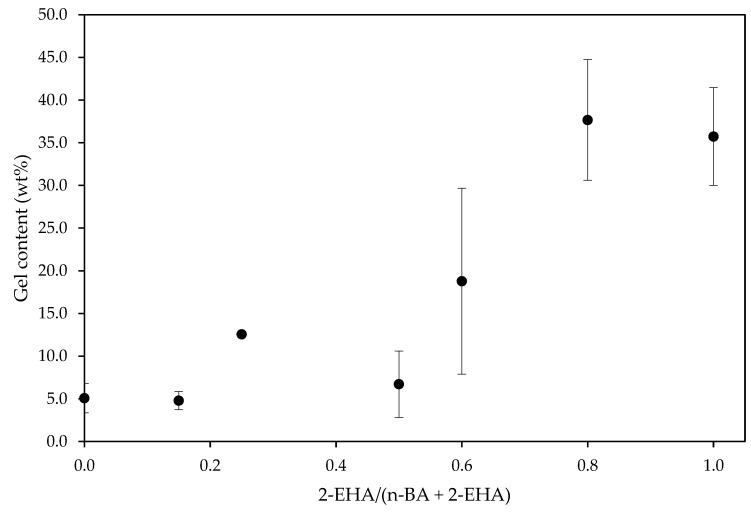
Variation of the gel content with the co-monomer ratio.

**Figure 3:**
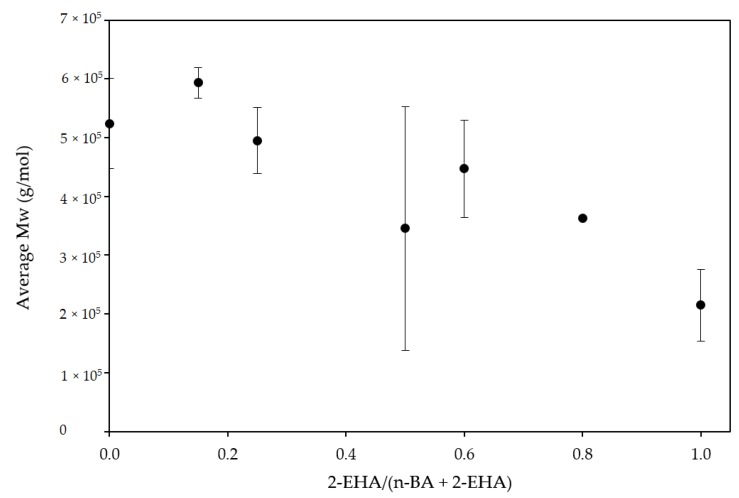
Influence of co-monomer ratio on the average sol molecular weight of the polymers produced.

The gel content and the average sol molecular weight were studied. The gel content grew with the increase of 2-EHA (Figure 2). This indicated that the chain transfer to polymer produced in the free radical polymerization of 2-EHA monomer was higher than with n-BA monomer. This affects the mobility of the polymer chains, which will be reflected in its adhesive properties and is in agreement with previous studies published [20,21,22]. As a consequence, the average sol molecular weight of the polymer decreased (Figure 3), since most of the polymer macromolecules formed were large enough to be insoluble in THF at 70 °C (higher than 7 × 10^6^ g/mol according to the literature) [23]. 

The formation of gel during emulsion polymerization, in absence of a crosslinking agent, often occurs by a intermolecular chain transfer to polymer followed by termination by combination. To form this insoluble fraction, it is necessary that long chain branches are produced. Another way to generate branching is by intramolecular chain transfer (backbiting). Some studies have shown that the intramolecular chain transfer reactions predominate over intermolecular chain transfer reactions [5,23,24]. Both generate tertiary radical species. However, the propagation of these radicals occurs at a very slow rate compared to the propagation of secondary radicals located at the end of the polymer backbone [25]. The intramolecular chain transfer to polymer generates short chain polymer branching and, in comparison to the intermolecular transfer, it does not provide a very significant contribution [22,26]. It must be taken into consideration that all polymerizations were carried out at the same temperature and had the same content of chain transfer agent, the tert-dodecyl-mercaptan, in the formulations. Without this agent, the gel content would have been higher. 

**Figure 4:**
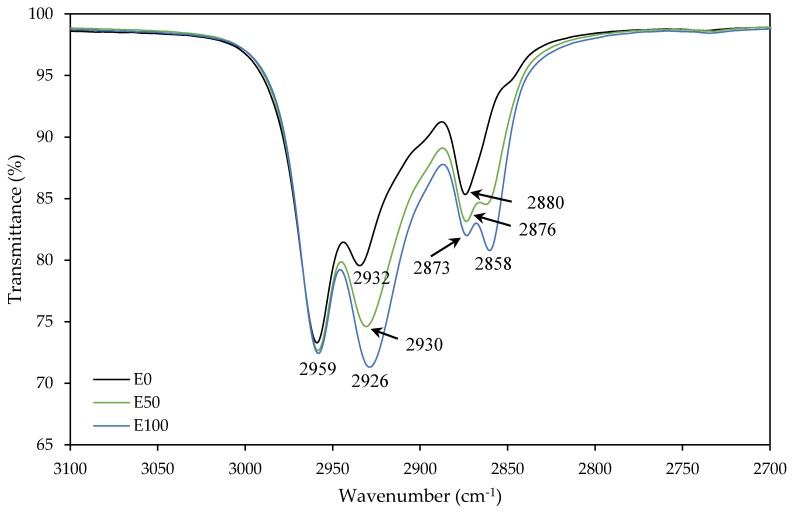
FTIR spectra of E0, E50 and E100 samples over a range of wavenumbers of 3100–2700 cm^−1^.

**Figure 5:**
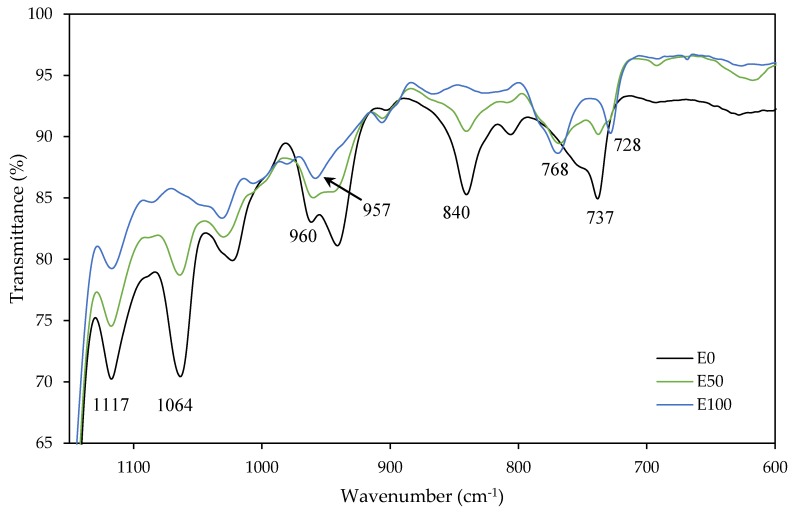
FTIR spectra of E0, E50 and E100 samples over a range of wavenumbers of 1150–600 cm^−1^.

To verify that both co-monomers had participated in the polymerization, the samples were analyzed by FTIR. Figure 4 and Figure 5 show changes in some characteristic bands according to the ratio 2-EHA/n-BA. The bands at 2959 cm^−1^, 2932 cm^−1^ and 2873 cm^−1^ were due to C–H stretching vibration. The band at 2932 cm^−1^ raised its intensity and was moved to 2926 cm^−1^ with the increase in the 2-EHA comonomer. Due to this, the singlet at 2873 cm^−1^ raised its intensity and become in a doublet with the appearance of a band at 2858 cm^−1^. The band at 1730 cm^−1^, characteristic from acrylic polymers, correspond to the C=O stretching of ester group. The peaks at 1264 cm^−1^ and 1158 cm^−1^ are due to the C–O stretching vibration of the saturated ester group. With the rise of 2-EHA, the band at 1117 cm^−1^ and 1064 cm^−1^, corresponding to C–C–C deformation of tertiary carbon, were disappearing. The same happened at 961 cm^−1^, corresponding to C–O–C deformation. In this case, the polymer with more n-BA co-monomer presented a band in each frequency, but the band at 942 cm^−1^ was disappearing with the increase of 2-EHA proportion and only the band at 958 cm^−1^ remained, decreasing its intensity. The band at 840 cm^−1^, corresponding to C–H deformation of the carbon near the oxygen, was disappearing. However, the band at 737 cm^−1^, corresponding to bond C–C of –(CH_2_)_3_–CH_3_ deformations of n-BA, disappear and become a doublet with the increase of 2-EHA at 768 cm^−1^ and 728 cm^−1^, corresponding to –C–(CH_2_–CH_3_)_2_ of 2-EHA monomer. Those ones corresponding to the rocking vibration of the carbons bonded to the tertiary carbon of 2-EHA monomer (assigned to the CH_2_ deformations of the alkyl sides) [10,27,28,29]. 

**Figure 6:**
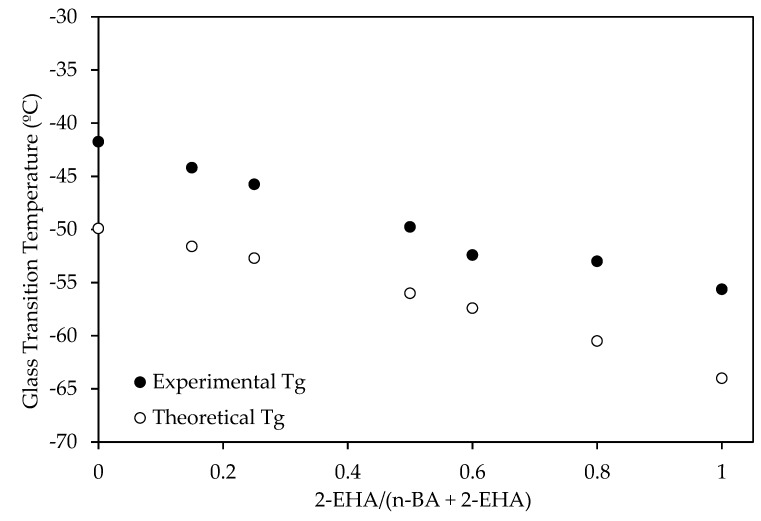
Influence of co-monomer ratio on the *T*_g_ values.

The *T*_g_ values obtained from the Fox equation and from the DSC experiments are comparatively shown in Figure 6. The observed decreasing trend of the *T*_g_ with the increase of 2-EHA was mainly attributed to differences in the chemical structure of these two monomers because the 2-EHA monomer is more flexible than the n-BA monomer [9,30]. 

The differences between the theoretical and experimental values were due to the fact that Fox’s equation assumes a two-component system composed by pure components and because the mixture is uniform [14].

### 3.2. Adhesive Properties 

**Figure 7:**
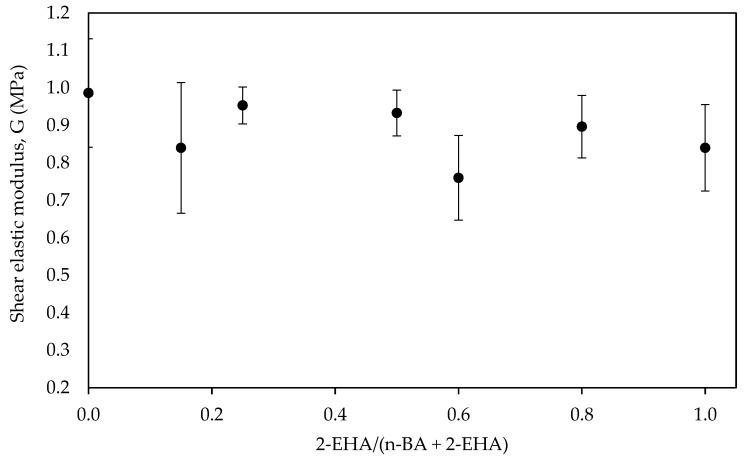
Shear elastic modulus versus the co-monomer ratio.

The shear elastic modulus (G) decreased slightly with the increase of the comonomer 2-EHA (Figure 7), as expected from its higher flexibility. This is most likely due to a lower *T*_g_ and to the fact that 2-EHA monomer has a longer side chain, which will lead a longer branch that will provide a lower polymer entanglement [31]. 

**Figure 8:**
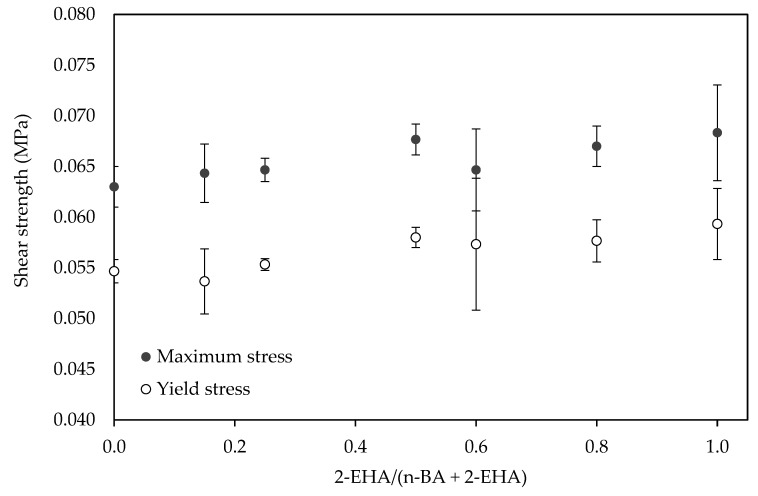
The effect of co-monomer ratio on the shear strength of the adhesives.

On the other hand, both the shear yield stress and maximum stress going up slightly with the increase of the 2-EHA, as shown in Figure 8. 

Two factors would be affecting on the values of shear properties. On one hand, increasing the 2-EHA amount decreases the *T*_g_, making the adhesive more flexible. On the other hand, the higher 2-EHA amount, the higher the gel content from a more crosslinked polymer (Figure 2), thus enhancing the cohesive strength and contributing to increasing the shear strength of the adhesive (Figure 8). The increase in gel content promoted a decrease in the other adhesion properties (i.e., peel and tack) as the balance between flexibility and cohesive strength is changed [32]. 

**Figure 9:**
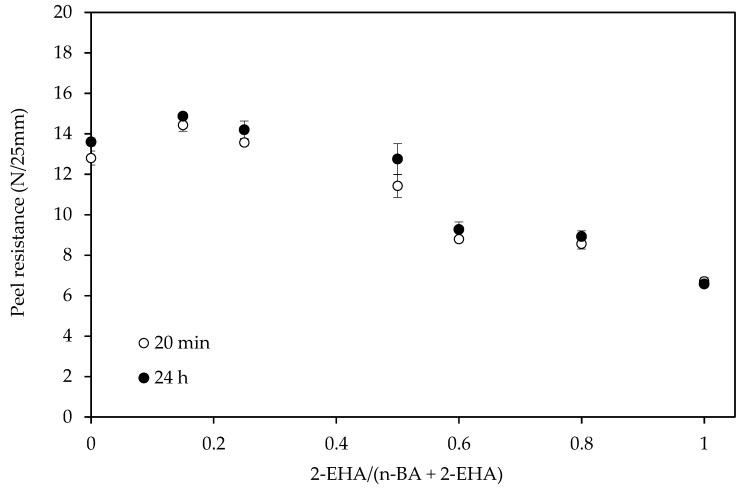
The effect of co-monomer ratio in the peel test using tapes of PET on glass panels (25 g/m^2^ of adhesive).

**Figure 10:**
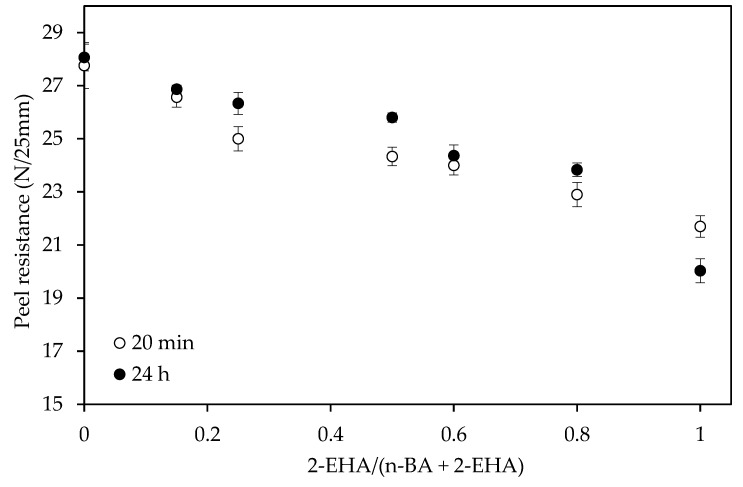
The effect of co-monomer ratio in the peel test using tapes of paper on glass panels (12 g/m^2^ of adhesive).

The effect of monomer composition on the peel resistance is shown in Figure 9 and Figure 10. The peel force values decreased with the increase of 2-EHA for both the PET and paper substrates used in this test.

As expected, the values found after 24 h were higher than the values recorded after a 20 min. When the peel test was carried out using 25 g/m^2^ of adhesive on PET substrate onto glass panel, cohesive failure were obtained in all cases. In the case of the paper substrate on glass panels, using 25 g/m^2^ of adhesive, structural failures were obtained at 20 min and 24 h, thus complying with the cellar requirements that the adhesive must be capable of breaking the paper to remove the label from the bottle.

In order to analyze and quantify the effect of the increase of 2-EHA comonomer in the peel resistance on paper substrate, 12 g/m^2^ of adhesive were used instead of 25 g/m^2^. Results showed (Figure 10) that the peel resistance with paper was higher than with PET even using a lower coating weight. The interaction between paper-adhesive and PET-adhesive is different. As the paper is a porous substrate, the adhesive penetrates into the paper resulting on a higher anchorage than in the case of PET, which is not a porous substrate. Moreover, the permanent deformation of the paper during the test is part of the fracture energy of the process. However, the PET is entirely elastic. For this reason, the paper tape, even with a thinner adhesive layer, showed higher peel resistance values than PET tapes.

**Figure 11:**
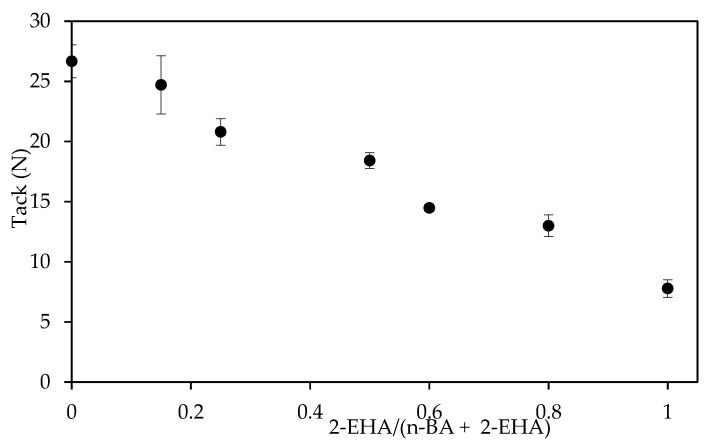
The effect of the co-monomer ratio in the loop tack test using PET tapes on glass panels.

The tack decreased as the content of 2-EHA increased, as shown in Figure 11. On one hand, increasing the amount of 2-EHA produced lower *T*_g_ polymers, which should increase the tack. On the other hand, increasing the gel content (Figure 2) resulted in higher cohesive strength, which counterbalances the adhesive properties and results in lower tack values [32]. The results suggest that, in this case, the second effect is more significant, probably because the *T*_g_ values are already very low in both cases. 

### 3.3. Water Resistance

**Figure 12:**
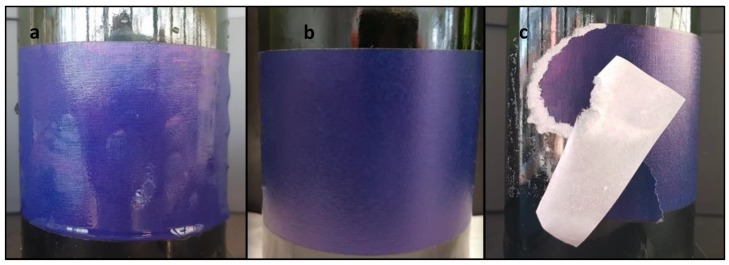
Optical pictures of the different states of a label of a same bottle using the adhesive E100 in the ice bucket test: (**a**) label just after remove the bottle from the ice bucket, (**b**) dried label after 24 h to remove the bottle from the ice bucket and (**c**) structural failure upon peel off the label manually.

To determine the water resistance of the adhesives in wet and cold conditions, the ice bucket test was carried out. All labels behaved in the same way. After 24 h submerged in the water bath the labels remained stuck with some wrinkles on the edges of the label. 24 h after removing the bottle from the ice bucket, with the labels dried, the wrinkles disappeared. When labels were removed from the bottle surface manually, structural failures occurred as shown in Figure 12, thus complying with the cellar requirements.

## 4. Conclusions

A set of acrylic PSAs were prepared by using emulsion polymerization, changing the ratio of 2-EHA/n-BA. The increase in the content of 2-EHA comonomer resulted in a lower *T*_g_ of the final polymer. As a consequence, the shear elastic modulus decreased slightly. The 2-EHA comonomer promoted gel formation, through intermolecular chain transfer to polymer followed by means of termination by combination. This was observed to happen to a higher extent with 2-EHA than with n-BA. However, the average sol molecular weight decreased, as most of the chains rich in 2-EHA monomer were too crosslinked to dissolve in THF at 70 °C and became part of the gel fraction. Both shear yield strength and maximum shear stress, determined by dynamic shear test, increased as a result of the increase in gel content. As expected from adhesives with higher cohesive strength, both peel resistance and tack decreased. In the peel resistance test, on paper substrates, a structural failure resulted in all cases. In addition, all labels showed an excellent water resistance in the ice bucket test. Thus, these adhesives meet the usual requirements of wine cellars.

## Figures and Tables

**Table 1:** Monomer composition (phm) of the adhesives.

Sample	n-BA	2-EHA	AA
E0	97.5	0.0	2.5
E15	82.9	14.6	2.5
E25	73.1	24.4	2.5
E50	48.8	48.8	2.5
E60	39.0	58.5	2.5
E80	19.5	78.0	2.5
E100	0.0	97.5	2.5

**Table 2:** Particle size and viscosity values of the synthesized acrylic Pressure-Sensitive Adhesives (PSAs), pH = 7.5 and 50 wt.% of solids in all cases.

Sample	Average Particle size (nm)	Viscosity (cP)
E0	191 ± 1	95 ± 2
E15	191 ± 1	110 ± 11
E25	221 ± 3	103 ± 16
E50	175 ± 1	163 ± 17
E60	185 ± 2	148 ± 3
E80	189 ± 1	153 ± 6
E100	186 ± 1	250 ± 15
